# Effects of EEG examination and ABA-therapy on resting-state EEG in children with low-functioning autism

**DOI:** 10.3934/Neuroscience.2020011

**Published:** 2020-06-05

**Authors:** Galina V. Portnova, Oxana Ivanova, Elena V. Proskurnina

**Affiliations:** 1 Institute of Higher Nervous Activity and Neurophysiology of RAS, 5A Butlerova St., Moscow 117485, Russia; 2 FSBI Federal medical center Rosimushchestvo, 31 Kalanchevskaya str., 107078, Moscow, Russia; 3 Research Centre for Medical Genetics, 1 Moskvorechye St., Moscow 115522, Russia

**Keywords:** EEG, low functioning autism, tactile defensiveness, applied behavior analysis, resting state, microstate

## Abstract

**Objective:**

We aimed to study the effects of EEG examination and ABA-therapy on resting-state EEG in children with low-functioning autism and tactile defensiveness.

**Methods:**

We have performed this study with three cohorts of preschoolers: children with autistic spectrum disorder (ASD) who needed applied behavior analysis (ABA) therapy due to their tactile defensiveness; children with ASD who didn't need ABA therapy; and the control group of healthy children. Number of microstates was determined in the initial and final parts of the resting-state EEGs.

**Results and conclusions:**

Children with higher tactile defensiveness for the most part had specific EEG microstates associated with unpleasant emotions and senses. The EEG microstates of children with ASD who did not need ABA therapy, had more similarities with the EEG microstates of typically developing children except for temporary changes. Meanwhile, the children with tactile defensiveness demonstrated typical patterns of EEG microstates from start to finish of the procedure.

## 1. Introduction

The study of cognitive and mental activity in subjects with low-functioning autism is very important for understanding the pathogenesis of the disease. However, such research meets certain difficulties. Among others, low-functioning autists are usually unable to follow instructions, do not let anyone touch them; they may be aggressive or demonstrate unreasonable behavioral reactions that are difficult to explain [1]. Among the methods of neuro visualization, the electroencephalography (EEG) has undoubted advantages when examining low-functioning autists due to its portability and good temporal resolution. However, before EEG testing, subjects should do some actions such as putting on an EEG electrode cap with adjusting the electrodes and following several elementary instructions [2]. However, children with autism spectrum disorder (ASD) have a limited ability to explore their environment regardless of the severity of their ASD. Such limitations result in other impairments which aggravate environmental communication and overall quality of life. Even patients with high-functioning autists have problems with interpretation of social cues because it is difficult for them to identify the emotional state of other person by speech traits [3] or voice pitch [4]. At the same time, the behavior of low-functioning autists could be described by specific symptoms including severe deficits in communication skills, aggressiveness and bizarre or self-injurious behavior that were not detected in their high-functioning peers [5].

For example, both groups had impairments in simultaneous perception [6], and consequently displayed a lack of understanding of social contexts and communication disruptions. Other characteristics displayed by both groups include narrow interests in and deficiencies in communication and social ability [7]. The most important difference between low- and high-functioning autism lies in the impairment of auditory and speech perception [8]. Moreover, the evidence of motor impairment in low-functioning subjects was found [9]. To summarize, despite some similarities in symptoms with regards to cognitive and mental impairment in low- and high-functioning groups, it is not obvious that both groups had the same nosology and pathogeneses [1],[10]. As a result, further work with patients who have low-functioning autism should be of a great importance.

There are few neuroimaging studies made with the participation of low-functioning autists. Most of these studies were based on a simple paradigm or focused on the research of resting state [11] or a passive presentation of stimuli [12]. Because of the difficulties related to medical procedures for children with low-functioning autism, these patients may not receive sometimes necessary medical treatment or even acute care [13].

Besides the behavioral disturbances that are typical for most children with impaired development, the tactile hypersensitivity and intolerance to touch could be considered as a specific trait of ASD, which hampers EEG examination [14]. The attempts to perform EEG in low-functioning autists who suffered from the hypersensitivity of the head, ears, or neck induced an extremely negative response of the children and made the procedure impossible [15]. Preparing for medical and experimental procedures could be one of possible solutions to this problem. Applied behavioral analysis (ABA) was previously used as a method of behavior correction in children with ASD to improve social skills, communication, and learning skills through positive reinforcement. This method was also used for variable interventions using a systematic desensitization procedure [16],[17]. The advantage of this method lies in the possibility of influencing a child's particular skill and the continuous collection of the behavioral data [18].

The resting state EEG is the most accessible method for research of mental states in children with low-functioning autism, because it doesn't require to understand and follow complex instructions and to perform cognitive tasks [19],[20]. Moreover, the resting states research allowed real-time investigating the natural dynamics of the child's mental activity. Among the EEG methods of analysis, we considered the methods sensitive to the temporal characteristics of the stable states of EEG, which could explain the behavioral responses of children with ASD.

Among the methods applied in resting state EEG studies, the microstate method [21] allowed to identify the series of quasi-stable states of EEE that were characterized by a unique topography and frequency. The microstate analysis took into account both the temporal and topographic characteristics of the EEG and therefore had a number of advantages compared to methods which were based on calculated averaged EEG values. In particularly, previous findings showed the absence of significant differences in the resting states averaged EEG parameters between typically developing children and children with ASD which were approximately the same age [22] or level of intellectual development [23].

Here, we aimed to examine the differences of the resting-state EEGs between cohorts of children with low-functioning autism who received or did not receive ABA therapy and typically developing peers. At the same time, we took into account two assumptions: first, ABA therapy could influence on cognitive or mental states of children with ASD compared to the individuals with ASD who get it for the first time. They were not familiar with the EEG procedure and were scared by the examination. Second, we hypothesized that it was the tactile defensiveness which interfere the EEG procedure. It resulted in unpleasant sensations which remained even after ABA therapy and should be considered in our study.

## 2. Methods

### 2.1. Participants

The prospective controlled trial was performed in 2016–2018 at the Center for Children with Autism, where children with ASD were examined with EEG. During the study, these patients received an applied behavior analysis (ABA)-based intervention program in the case of difficulties.

The inclusion criteria for ASD groups were as follows: an autism diagnosis based on the ICD-10 Criteria (F84.0) and the Child Autism Rating Scale (CARS > 35). The Autism Diagnostic Observation Schedule (ADOS-2) with cut-off score 10 was used for children between the ages of three and five years old. The inclusion criteria for the control cohort were as follows: the Child Autism Rating Scale (CARS) less than 30, with the ADOS-2 out of specter, the ages between three and five years old.

The exclusion criteria for all groups were as follows: children with disorders other than autism, no antipsychotic drugs or other medical therapy, IQ higher than 70 according to Wechsler Preschool and Primary Scale of Intelligence (WPPSI), see Table 1.

**Table 1.** Descriptive statistics of age and results of parents' interview.

	Age	ADOS-2	CARS	non-verbal scale of WPPSI	Tactile Hypersensitivity scores
ASD group	4.1 ± 1.2	13.9 ± 3.8	43.8 ± 6.8	101.6 ± 9.9	4.3 ± 2.6
ASD + ABA group	3.9 ± 1.1	14.5 ± 3.7	45.2 ± 7.1	100.7 ± 6.1	10.8 ± 1.8
Control group	4.0 ± 0.9	2.7 ± 1.5	23 ± 5	104.6 ± 7.3	0.9 ± 0.8

#### 2.1.1. Three groups of subjects participated in our study

ASD + ABA group: 10 low-functioning autists from 3 to 5 years old (3.9 ± 1.1 y.o., 45 ± 5 scores by CARS, moderate or severe level of autism by ADOS-2) underwent ABA therapy. The children in this group, due to their aggressiveness, destructive behavior, and primarily tactile hypersensitivity, could not get the EEG examination on their first attempt. Difficulties were related primarily to hypersensitivity of the head, ears, or neck. When they did not allow touching of the head, ears or neck, it was because they did not want to put on a cap or wash off the cat hair. The other reason was related to behavior disorders: the children reacted negatively to the strangers, did not follow instructions, cried or screamed, refused to enter the room, or were physically resisted.

ASD group: 25 low-functioning autists 3–5 years old (4.1 ± 1.2 y.o., 43 ± 6 scores by CARS, moderate or severe level of autism by ADOS-2), who were successfully investigated on the first attempt. Most children in this group also showed the features of behavior disorders; however, they did not show symptoms of tactile defensiveness and were persuaded or held during the examination.

Control group consisted of 30 children 3–5 years old (4.0 ± 0.9 y.o., 23 ± 5 scores by CARS, out of RAS by ADOS-2).

### 2.2. ABA procedure

The children received ABA training at the Center for Disabled Children. They received at least three hours of discrete trial training weekly and attended classes from one to three times a week. The duration of each lesson was 30 minutes, and the method of systematic desensitization was used [24],[25]. In this program, an ABA-certified therapist worked one-on-one to teach functions such as imitation, cooperation, and speech. Based on ABA principles and procedures, the training also included various exercises designed to improve a child's weakest areas to help to develop coordination and movement. The children with no speech ability were trained to use the Picture Exchange System to communicate with others.

The number of lessons required for a successful EEG recording was determined individually and ranged from three to 16 (on average, 6.5 lessons). The ABA therapy was successful if the following criteria were met:

(1) The child could sit quietly for 25 minutes with an EEG cap on his head with the electrodes and EEG gel.

(2) The child could calmly react to the stimuli of various modalities during functional testing.

When the ABA therapy was successfully finished, we attempted to finish study (only one attempt to register EEG).

### 2.3. EEG procedure

The EEG recording procedure was carried out by the EEG specialist and ABA-certified therapist (during EEG recording and putting on the EEG cap). Both specialists also participated in EEG studies performed with the other children. The EEG section consisted of three stages: (1) resting-state EEG at the beginning of the examination, (2) hyperventilation and photic stimulation, and (3) the resting-state EEG at the end of the session. The EEG examination involved a three-minute resting state EEG with the patients' eyes opened at the beginning of the study, a series of functional clinical tests (photic stimulation and hyperventilation), a resting-state EEG with the patients' eyes closed, and a three-minute resting-state EEG with the patients' eyes opened at the end of the examination.

### 2.4. Behavioral assessments

The behavioral responses were analyzed from the beginning to the end of the study. The behavioral and emotional responses were registered using video camera Samsung HMX-F90 and assessed by two experts. We assessed the symptoms of nervous behaviors (fidgeting, thumb sucking, body rocking); episodes of crying and whimpering or smiling and laughter; and symptoms of relaxation and tiredness (motor activity and relaxation poses). The symptoms of hypersensitivity (tactile, acoustic, or visual) and behavior disorders were fixed during the diagnostic procedure and used when selecting groups of the children.

The tactile defensiveness was assessed basing on the test described in Supplementary Information [26],[27]. Using this test, we calculated scores of tactile defensiveness (0–15 scores) according to the parents' reports. Children of control group had 0.9 ± 0.8 scores, ASD + ABA group had 10.8 ± 1.8 scores and ASD group 4.3 ± 2.6 scores. All interviews of parents as well as CARS, ADOS-2 and WPPSI testing were recruited within a week before the study

### 2.5. EEG recordings

We recorded the resting-state EEGs (sampling rate of 250 Hz) for 15 minutes using EEG amplifier “Encephalan” (Medicom MTD, Taganrog, Russian Federation) with 19 AgCl electrodes placed according to the International 10–20 System. The electrodes placed on the left and right mastoids served as joint references under a unipolar montage. The vertical electrooculogram (EOG) was measured with AgCl cup electrodes placed 1 cm above and below the left eye canthus. The horizontal EOG was measured with electrodes placed 1 cm lateral from the outer canthi of both eyes. The electrode impedances were less than 10 kΩ.

### 2.6. Data reduction

EEG data were digitally filtered (2–30 Hz) and re-referenced using an average reference that was applied after having excluded channels near the eyes. Data were then exported to Matlab 7.6 and were visually inspected using EEGLAB data. Movements and electrical artifacts were removed. Data segments were a minimum of 30 seconds long for inclusion in further analysis. The average segment length of usable data for the control group was 215 s, 214 s for group with ASD + ABA and 203 s for the ASD group. The eight 12-second EEG fragments (the minimal duration in any group) from the beginning to the end of the EEG study were chosen for further microstate analysis.

### 2.7. Data analysis: Power Spectral Density (PSD)

Fast Fourier transform was used to analyze PSD using open source code in the Matlab Platform (MathWorks). The resulting normalized spectra were integrated over intervals of unit width in the range of interest (2–3 Hz, 3–4 Hz, … 29–30 Hz). The bands we analyzed were defined as follows: delta (2–4 Hz), theta (4–8 Hz), alpha (8–12 Hz), beta1 (12–20 Hz Hz), beta2 (20–30 Hz).

### 2.8. Data analysis: microstate analysis

The EEG microstate analysis was used to investigate the spatio-temporal features of the rsEEG [28]. The microstate segmentation was made separately for each group and the condition (the first part or the last part of the EEG registration). According to the optimized iteration scheme results, the three clusters were selected after k-mean clustering. The extraction of GFP peak maps was calculated using 1000 GFP peaks per subjects with a minimum peak distance of 10 ms. A modified K-means algorithm was used to segment EEG data into microstates. Smoothing was made by rejecting segments smaller than 100 ms.

### 2.9. Statistical analysis

Clusters' allocation with their metrics (duration, localization) was carried out according to the standard algorithm using algorithm from the EEGLAB toolbox [28]. The correlation analysis was made between individual data for each cluster received using the EEG microstate analysis, as well as individual PSD data in the range of interest. This was completed using Spearman's rank correlation for areas of interest (LF: F3–P4, RF: F4–P3, P–(P3+P4)–(F3+F4), F–(F3+F4)–(P3+P4)). Only the significant results (after the Bonferroni correction, p < 0.05) were described further.

A one way and the repeated measures ANOVA with Bonferroni correction for multiple comparison (p < 0.05), was completed to determine group effect and temporal effect (from the beginning to the end of the study) on EEG metrics (PSD, durations of EEG microstates). Group effects for behavioral tests were also analyzed using ANOVA grouping factor and Mann-Whitney U-test. The correlation analysis was made using Spearman Rank correlation to examine the relations between EEG metrics and behavioral tests (the scores of tactile hypersensitivity and other behavioral results).

## 3. Results

### 3.1. Behavioral assessments

**Figure 1.**
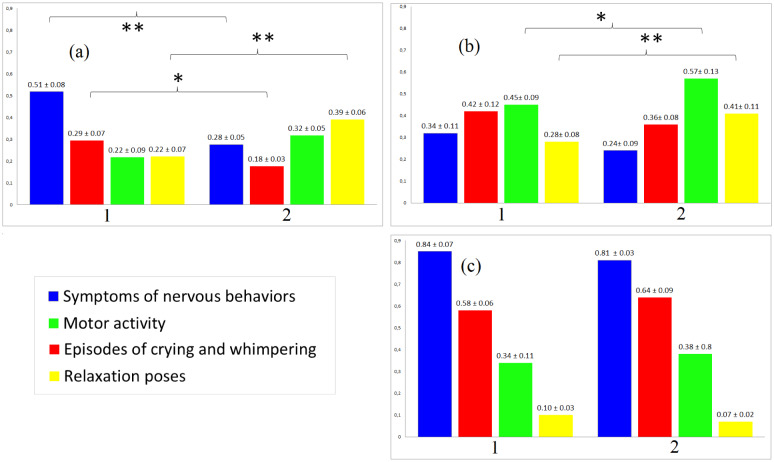
Scores behavioral responses during the EEG study that were calculated for each 8 12-sec intervals (one score = one behavioral response of each type) and then averaged separately for the beginning and of the study. 1—beginning of the study, 2—end of the study. (a)—control group of subjects, (b)—ASD + ABA group (c)—ASD group.

The behavioral analysis showed that the control group and the ASD + ABA group had less symptoms of nervous behaviors and episodes of crying and whimpering. Further, the control group and the ASD + ABA group displayed behavioral dynamics during the EEG study. At the end of the study they demonstrated symptoms of relaxation and tiredness (motor activity and relaxation poses). The children of the ASD group did not show typical behavioral dynamics during the EEG study (Figure 1).

### 3.2. Power spectral density (PSD)

The group differences between PSD in 2–14 Hz were not found. The beta-rhythm power 12–20 Hz in the first part and at the end of the study was significantly higher in the ASD group (F(2, 61) = 5.6782, *p* = 0.008103) in frontal areas (Table 2).

**Table 2.** Mean ± SD for PSD in bands of interest in frontal (F3, F4) and parietal (P3, P4) regions. Bold font means significant group differences.

Sessions of study	Group	Area	Delta (2–4 Hz)	Theta (4–8 Hz)	Alpha (8–12 Hz)	Beta1 (12–20 Hz Hz)	Beta2 (20–30 Hz)
Beginning of the study	Control group	Parietal	33.2 ± 7.0	18.3 ± 5.2	22.9 ± 9.9	4.2 ± 4.9	3.5 ± 2.8
		Frontal	29.9 ± 6.8	17.9 ± 5.6	17.3 ± 7.4	5.3 ± 7.1	4.0 ± 5.2
	ASD +ABA group	Parietal	35.5 ± 7.9	16.8 ± 7.4	19.1 ± 10.5	5.6 ± 7.4	3.8 ± 4.5
		Frontal	32.8 ± 8.3	17.2 ± 6.8	13.8 ± 9.3	5.0 ± 6.2	4.1 ± 5.3
	ASD group	Parietal	36.1 ± 8.2	19.1 ± 4.7	18.9 ± 8.1	5.8 ± 5.5	4.0 ± 4.6
		Frontal	37.0 ± 6.9	18.0 ± 5.9	12.8 ± 11.6	**7.9 ± 10.3**	2.9 ± 3.4
End of the study	Control group	Parietal	34.1 ± 6.6	17.9 ± 4.8	21.7 ± 8.5	4.8± 6.6	3.4 ± 4.9
		Frontal	30.7 ± 6.8	17.3 ± 5.8	17.9 ± 7.4	5.8 ± 7.9	3.9 ± 5.2
	ASD +ABA group	Parietal	35.9 ± 5.7	19.1 ± 7.4	18.8 ± 8.9	5.3 ± 6.3	3.7 ± 5.9
		Frontal	36.7 ± 5.9	18.2 ± 7.1	14.2 ± 8.5	5.2 ± 6.5	4.2 ± 6.1
	ASD group	Parietal	33.7 ± 8.4	18.5 ± 6.3	18.8 ± 7.3	6.3 ± 6.0	3.8 ± 5.2
		Frontal	36.6 ± 7.9	18.7 ± 6.8	13.4 ± 9.5	**8.2 ± 9.1**	3.1 ± 5.9

### 3.3. Microstates at the first part of the study (Figure 2)

**Figure 2.**
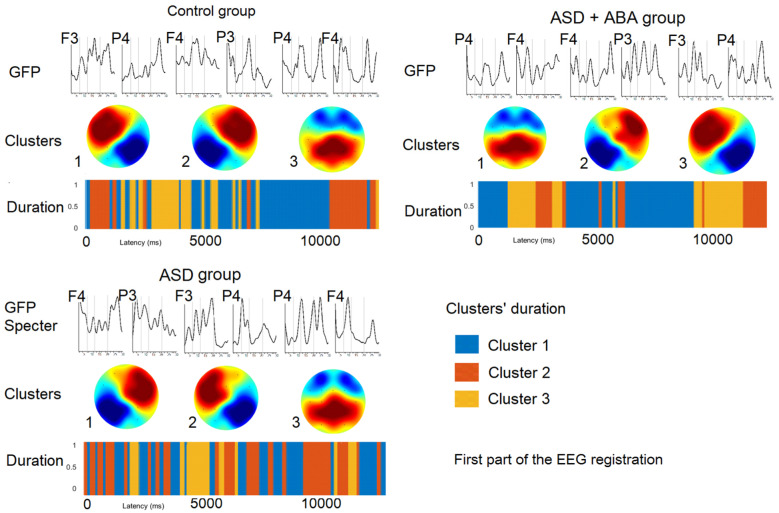
The results of EEG microstates analysis prepared on the first EEG fragment for each group separately. GFP—Global field power.

The similar microstate clusters were detected in different groups of children, their time of presence were represented in Table 3.

**Table 3.** Time of presence of each cluster (sec).

Group	Duration of cluster (sec)							
	Beginning of the study				End of the study			
	LF	RF	P	F	LF	RF	P	F
Control group	6.11	3.24	2.65	-	6.93	4.79	-	0.29
ASD + ABA group	3.72	2.81	5.47	-	-	3.35	5.81	2.84
ASD group	5.10	5.17	1.72	-	6.06	5.39	0.56	-

The resting state EEG microstates in children of control group at the first part of the study had left-frontal to right-posterior cluster (LF), right-frontal to left-posterior cluster (RF) and mostly parietal cluster (P). The resting-state EEG microstates of children with tactile hypersensitivity (ASD + ABA group) had mostly parietal cluster (P), right-frontal to left-posterior cluster (RF), left-frontal to right-posterior cluster (LF). EEG microstates of children with ASD who were not required to get ABA therapy (ASD group) had right-frontal to left-posterior cluster (RF), left-frontal to right-posterior cluster (LF) and mostly parietal cluster (P).

### 3.4. Microstates at the last part of the study (Figure 3)

**Figure 3.**
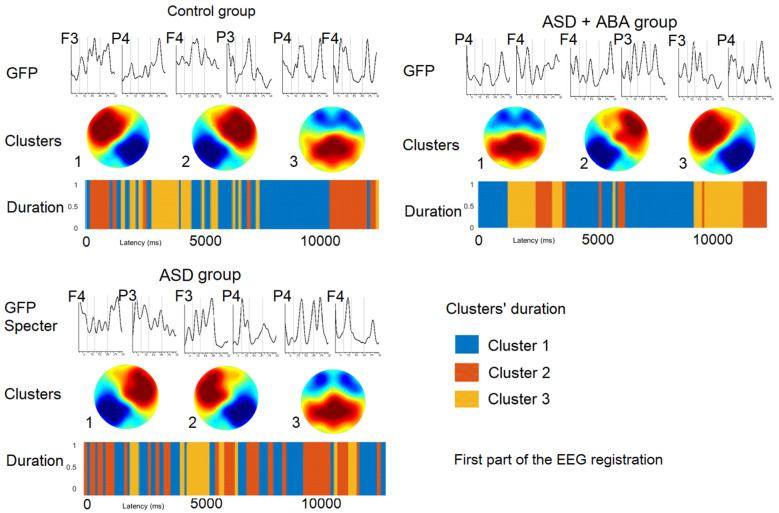
The results of EEG microstates analysis prepared on the last EEG fragment for each group separately. GFP—Global field power.

The resting state EEG microstates in children of the control group at the first part of the study had left-frontal to right-posterior cluster (LF), right-frontal to left-posterior cluster (RF) and mostly frontal cluster (F). The resting state EEG microstates of ASD + ABA group had mostly parietal cluster (P), right-frontal to left-posterior cluster (RF) and mostly frontal cluster (F). EEG microstates of children of ASD group had left-frontal to right-posterior cluster (LF), right-frontal to left-posterior cluster (RF) and mostly parietal cluster (P).

### 3.5. Results of correlation analysis

Cluster LF (left-frontal to right-posterior) positively correlated with the beta-band (12–20 Hz; *r* = 0.54, *p* = 0.009). Cluster RF (right-frontal to left-posterior) negatively correlated with the alpha-band (7–11 Hz; *r* = −0.61, *p* = 0.006) and beta-band (12–20 Hz; *r* = −0.52, *p* = 0.01). Cluster P (mostly parietal) negatively correlated with the alpha-band (7–11 Hz; *r* = −0.54, *p* = 0.01). Cluster F (mostly frontal) positively correlated with the theta-band (4–7 Hz; *r* = 0.67, *p* = 0.003) and alpha-band (7–11 Hz; *r* = 0.51, *p* = 0.02).

**Figure 4.**
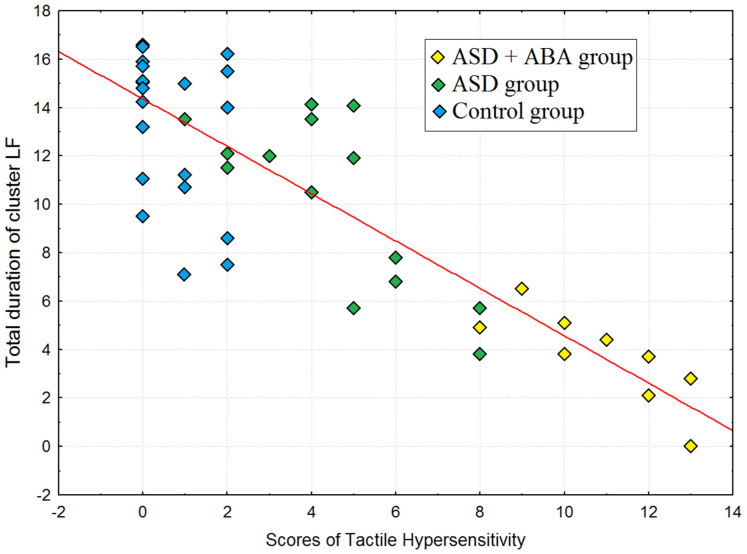
Scatterplot for correlation between total duration of cluster LF (the sum of duration of LF at the beginning of study and duration of LF at the end of the study) and scores of tactile hypersensitivity.

The symptoms of relaxations and tiredness correlated with the duration of the F cluster (over all groups) at the end of the study (*r* = 0.64, *p* = 0.005). The ratio between total duration of the LF cluster negatively correlated with symptoms of tactile hypersensitivity in subjects (*r* = −0.74, *p* = 0.0003), the results of the correlation analysis were depicted on Figure 4.

### 3.6. EEG dynamics

The average PSD did not differ significantly between fragments at the end and at the beginning of the study. The LF cluster was predominant in the control group, and it was significantly less presented in children with ASD, especially in the ASD + ABA group. Its time of presence increased in the ASD group (F(2, 61) = 5.127, *p* = 0.00993), from the beginning to the end of the study, compared to other groups of children. The RF cluster was found in all groups and the time of presence increased at the end of the study (F(2, 61) = 7.564, *p* = 0.00164). The P cluster was presented more during the first part of the study and decreased at the end of the study (F(2, 61) = 6.005, *p* = 0.00613). The F (mostly frontal) cluster was detected only in the control and the ASD + ABA groups and appeared only at the end of the study (F(2, 61) = 5.433, *p* = 0.00887).

## 4. Discussion

Our findings demonstrated that despite the averaged EEG features were similar in different group of children, they had different temporal characteristics of the EEG correlated with their behavioral responses. The previous studies showed just a few differences of the EEG's rhythmic activity between children with ASD and typically developing children of the same age, gender, and IQ [29]–[32]. The similar differences between the PSD in the control and the ASD groups were revealed in our study. At the same time, our findings demonstrated that in spite of similar spectral characteristics of the EEG, the dynamics of the EEG oscillations measured by the microstates methods [28],[33] and associated with conscious mind states [34], differed between the ASD and the typical groups [35],[36] and also depended on the time that elapsed from the beginning of the study.

Conscious mind state associated with the RF cluster was accompanied by an increase of the beta-band and a decrease of the alpha-band, which were previously associated with higher cortical activity [37],[38]. These states were prevalent in children for whom the situation of registering for the EEG was a new experience. The highest duration of RF cluster was found in children with ASD for whom the situation of EEG study was most unfamiliar compared to control group children and children with ASD received ABA therapy. These data were consistent with the previous findings demonstrated the higher presence of microstates associated with default mode network (DMN) in control group, compared to individuals with ASD who focused on inviroment than self-memory retrieval [35]. Thus, the higher frontal activity in the control and ASD groups could be associated with studying unfamiliar situations [39],[40]. At the same time, the duration of the LF cluster which was lowest in the ASD + ABA group and highest in control group, inversely correlated with symptoms of tactile hypersensitivity. According to the previous data, the similar parameter of predominance of the beta-rhythm wavelet transformation in the left hemisphere compared with the right was correlated with higher effective emotional processing [41]. Regarding negative emotions, other studies reported dominance in the right hemisphere and the left hemisphere [42]. So, we hypothesized that the children of the ASD + ABA group had a negative LF/RF ratio because they continued to experience negative emotions during procedure, but then followed these emotions with a skill learned from ABA therapy. Moreover, the cluster with centro-parietal alpha-rhythm desynchronization, previously related with pain or negative emotions [43],[44] was dominate in the EEG for the ASD + ABA group.

The cluster correlated with higher theta and alpha-bands and accompanied by behavior responses of fatigue and relaxedness was revealed at the end of the study in the control group, as well as for subjects of the ASD + ABA group. The described behavior dynamics are typical for the EEG studies [45] and indicated that the children had successfully adapted to the experimental situation. In contrast, the children of the ASD group did not show these behavioral dynamics during the EEG. They were the most likely to be stressed throughout the study.

To sum, we detected the most marked symptoms of tactile defensiveness in those children with RAS who could not pass through the EEG examination without preliminary therapy. However, tactile defensiveness is observed to varying degrees in all children with ASD, it may be one of the reasons for the disfunction of social communication with other people from the early childhood. In relation to this fact, of special importance is preliminary training of children with ASD with ABA therapy and other methods, which allows to prepare the children for medical examinations.

## 5. Conclusions

In summary, our study revealed that tactile hypersensitivity, which was the main symptom interfering with EEG procedure in children with ASD (ASD + ABA group) was associated with specific the brain activity during the EEG procedure and was accompanied by unpleasant feelings in spite of the ABA therapy took place. At the same time, the ABA method used to prepare for the EEG study enabled the EEG research, relieved the child's stress levels, contributed to the appearance of a typical for the control group behavioral and EEG dynamics during procedure and consequently should be further used.
