# Supplementary material for: Effects of EEG examination and ABA-therapy on resting-state EEG in children with low-functioning autism
Source: AIMS Neurosci. 2020 Jun 5;7(2):153–67. doi: 10.3934/Neuroscience.2020011 (PMC7321768; doi:10.3934/Neuroscience.2020011)
Supplement: Supplementary file 1 [file neurosci-07-02-011-s01.pdf]

AIMS Neuroscience
http://www.aimspress.com/journal/neuroscience

AIMS Neuroscience, 7(2): 153–167.
DOI: 10.3934/Neuroscience.2020011
Received: 05 March 2020
Accepted: 01 June 2020
Published: 05 June 2020

Review

Effects of EEG examination and ABA-therapy on resting-state EEG in children with low-functioning autism

Galina V. Portnova[1,*], Oxana Ivanova[2] and Elena V. Proskurnina[3]

[1] Institute of Higher Nervous Activity and Neurophysiology of RAS, 5A Butlerova St., Moscow 117485, Russia
[2] FSBI Federal medical center Rosimushchestvo, 31 Kalanchevskaya str., 107078, Moscow, Russia
[3] Research Centre for Medical Genetics, 1 Moskvorechye St., Moscow 115522, Russia

* **Correspondence:** Email: caviter@list.ru; Tel: +74953347000; Fax: +74997430056.

Supplementary Information

Our test was based on the Information for Parents and Professionals on Behavioral Indicators of Tactile Defensiveness (http://dyspraxiafoundation.org.uk/wp-content/uploads/2013/10/Tactile_Defensiveness.pdf):

1. Avoidance Responses to Touch Stimulation

• Avoidance of certain styles or textures of clothing (e.g. scratchy or rough); or conversely an unusual preference for certain styles or textures of clothing (e.g. soft materials, long sleeved shirts)
• Avoidance of contact with other children, e.g. preference for standing at the end of line during assembly, staying at the edge of a group during story time etc.
• Avoidance of anticipated touch or from interactions involving touch, e.g. tendency to pull away or avoidance of touch to the face
• Avoidance of play activities involving tactile materials (e.g. sand, finger paints) or body contact, with a tendency to prefer solitary play
• Avoidance of going barefoot, especially in sand and grass (could result in tip-toe walking)
• Avoidance of a crowded environment, likes to stay under the table, behind the settee or under the staircase

2. Aversive Responses to Non-Painful Touch

• Aversion or struggle when picked up, hugged or cuddled

• Aversion to certain daily living tasks, e.g. having showers, cutting fingernails and hair and face washing

• Aversion to dental care and/or brushing teeth

• Aversion to being handled during daily activities, e.g. changing nappy or clothes, cleaning nose or face

• Aversion to being approached from behind. May rub skin or scratch area being touched

3. Emotional Responses to Touch Stimulation

• Becomes anxious and distressed when being physically close to people, e.g. during assembly, inside the dining hall, etc.

• Refusal to participate in certain social activities, e.g. going to a party or supermarket

• Responding verbally or with physical aggression to light touch to arms, face or legs, e.g. lashing out

• Objection, withdrawal or negative responses to touch contact, including that encountered in the context of intimate relationship even in a friendly or affectionate manner. A tendency to prefer to touch rather than to be touched. Some children may seek firm touch in order to modulate the level of defensiveness.

AIMS Press

© 2020 the Author(s), licensee AIMS Press. This is an open access article distributed under the terms of the Creative Commons Attribution License (http://creativecommons.org/licenses/by/4.0)
